# The effect of SSRI/SNRI antidepressant treatment on the gut microbiota of patients with major depressive disorder

**DOI:** 10.1038/s43856-026-01782-5

**Published:** 2026-07-16

**Authors:** Eugenia Emile Natasha, Danique Mulder, Leon Fehse, Nils R. Winter, Lukas Fisch, Marius Welzel, Corinna Bang, Susanne Meinert, Kira Flinkenflügel, Tiana Borgers, Janik Goltermann, Elisabeth J. Leehr, Carsten Culmsee, Frederike Stein, Florian Thomas-Odenthal, Paula Usemann, Lea Teutenberg, Igor Nenadic, Benjamin Straube, Nina Alexander, Hamidreza Jamalabadi, Andreas Jansen, Robert Nitsch, Andreas Lügering, Andre Franke, Udo Dannlowski, Tilo Kircher, Dominik Heider, Tim Hahn, Janna N. Vrijsen, Philip van Eijndhoven, Indira Tendolkar, Andreas Reif, Sharmili Edwin Thanarajah, Silke Matura, Alejandro Arias Vasquez, Mirjam Bloemendaal

**Affiliations:** 1 Department of Psychiatry, Radboud University Medical Center, Donders Institute for Brain, Cognition and Behaviour, Geert Grooteplein Zuid 10, Nijmegen, The Netherlands; 2 University of Münster, Institute of Medical Informatics, Münster, Germany; 3 University of Münster, Institute for Translational Psychiatry, Münster, Germany; 4 Institute of Clinical Molecular Biology, Christian-Albrechts-University, Kiel, Germany; 5 University of Münster, Institute for Translational Neuroscience, Münster, Germany; 6 Center for Mind, Brain and Behavior (CMBB), Marburg, Germany; 7 Department of Pharmacy, Philipps-University Marburg, Marburg, Germany; 8 Department of Psychiatry and Psychotherapy, Marburg University, Marburg, Germany; 9 Medizinisches Versorgungszentrum Portal 10, Münster, Germany; 10 Bielefeld University, Medical School and University Medical Center OWL, Protestant Hospital of the Bethel Foundation, Department of Psychiatry, Bielefeld, Germany; 11 Depression Expertise Centre, Pro Persona Mental Health Care, Nijmegen, The Netherlands; 12 Behavioural Science Institute, Radboud University, Nijmegen, The Netherlands; 13 Department of Psychiatry, Psychosomatic Medicine and Psychotherapy, University Hospital Frankfurt – Goethe University, Frankfurt am Main, Germany; 14 Goethe University Frankfurt, Cooperative Brain Imaging Center - CoBIC, Frankfurt, Germany; 15 Fraunhofer Institute for Translational Medicine and Pharmacology, Frankfurt, Germany; 16 Department of Immunology, IfADo-Leibniz Research Centre for Working Environment and Human Factors, Dortmund, Germany; 17 Department of Internal Medicine, Erasmus Medical Center, Rotterdam, The Netherlands

**Keywords:** Depression, Psychiatric disorders

## Abstract

**Background:**

The gut microbiome has been linked to major depressive disorder (MDD), yet it remains unclear whether antidepressant treatment influences these associations. This study aimed to clarify the role of serotonin reuptake inhibitors (SSRI/SNRI) in shaping gut microbiome changes observed in MDD.

**Methods:**

We conducted cross-sectional analyses in two independent patient cohorts (total *N* = 1802) and a meta-analysis across both cohorts, comparing the gut microbiome of MDD patients with and without SSRI/SNRI treatment.

**Results:**

Here we show that SSRI/SNRI treatment is consistently associated with reduced *Clostridium sensu stricto 1* abundance. This effect is specific to SSRI/SNRI treatment and not observed with other psychotropic medications. Importantly, reductions in *Clostridium sensu stricto 1* in MDD compared to unaffected controls are explained by SSRI/SNRI medication status.

**Conclusions:**

Antidepressant treatment is an important factor shaping gut microbiome alterations linked to MDD, underscoring the need to account for medication effects and potentially informing future microbiome-based strategies to improve treatment response.

## Plain language summary

This study examined whether commonly used antidepressants (called SSRIs and SNRIs) influence the gut microbiome in people with depression. The gut microbiome is a community of bacteria in the intestines that may affect mental health. We analyzed data from more than 1,800 people, including individuals with depression who were taking or not taking SSRIs and SNRIs. We found that people using these medications consistently had lower levels of gut bacterium called *Clostridium* sensu *stricto 1*. This effect was not seen with other psychiatric medications. This means we identified a bacterium that is influenced by antidepressant use. In the future, this knowledge may help test whether the gut microbiome can improve treatment outcomes and support more personalized care.

## Abstract (web summary)

Natasha, Mulder et al. investigate gut microbiome in major depressive disorder to disentangle disorder-associated signals from the effects of antidepressants. SSRI and SNRI antidepressant use is associated with lower abundance of Clostridium sensu stricto 1, suggesting that part of the microbial signature of depression is driven by medication.

## Introduction

Major depressive disorder (MDD) is a debilitating condition that significantly reduces quality of life^1^. Despite its complex neurobiology, recent research highlights the gut microbiota as a potential contributor to MDD pathophysiology^2^. Microbial alterations may inform microbiota-targeting interventions to alleviate symptoms or improve treatment outcomes. However, findings on gut microbial changes in MDD have been inconsistent^3,4^, limiting identification of targetable features. These variations seem to arise partly due to within and between cohort variability in clinical presentation, host genetics, and environmental factors^5,6^. Another key source of variability is the use of psychotropic medications. Though psychotropics such as antidepressants and antipsychotics have demonstrated effects on the gut microbiota in animal models and in vitro, their importance has been underappreciated in clinical research^7–11^.

Psychotropic medication use is often inadequately controlled for in human cohort studies, due to limitations in clinical phenotyping and sample size. For example, population studies have examined the effects of antidepressants on the gut microbiome without considering disease status^12,13^. Furthermore, clinical cross-sectional studies often include both medicated and unmedicated patients without stratifying the analyses by medication status^10^, while longitudinal observational studies typically compare medicated patients only to unaffected individuals^14,15^. Additionally, psychotropic medications are frequently analyzed as a single group^16,17^, neglecting pharmacokinetic differences between drug classes that may result in varying effects on the gut microbiota^7,18,19^. Together, these designs limit insights into medication-specific effects.

In this work, using large clinical cohorts, we identify gut microbial genera whose associations with MDD differ depending on antidepressant treatment status, focusing on the most commonly prescribed antidepressant classes for MDD: Selective Serotonin and Norepinephrine Reuptake Inhibitors (SSRIs/SNRIs). In the discovery cohort (MACS, *n* = 1568), several MDD-associated genera show associations that are modified by SSRI/SNRI treatment, findings that are subsequently replicated in an independent cohort (MIND-Set, *n* = 234) and confirmed through meta-analysis. These results highlight SSRI/SNRI treatment as a modifier of the association between the gut microbiome and MDD, laying the groundwork for targeted studies investigating how antidepressant-induced microbial changes relate to treatment response and potentially informing microbiome-based therapeutic strategies in psychiatry.

## Methods

### Study populations

#### Cohort description

##### Marburg-Münster Affective Cohort Study (MACS) cohort

The MACS cohort^20^ is an observational, prospective, case-control study, conducted at the Department of Psychiatry and Psychotherapy at the University of Münster and University of Marburg, Germany. The study, described previously^20,21^, aims to investigate underlying neurobiological and environmental risk factors for affective disorders. The cohort comprises patients with an acute or lifetime diagnosis of an affective disorder and a group of unaffected individuals who reported no history of any psychiatric disorders. Individuals aged 18–67 years, of Caucasian ancestry, receiving in- or outpatient treatment from participating local clinics in Marburg or Münster, or responding to newspaper advertisements, were recruited between 2014 and 2018. Study procedures were conducted at the Marburg University Hospital and Münster University Hospital. Psychiatric diagnoses, including an MDD diagnosis, were determined by trained clinicians using the Structural Clinical Interview for DSM-IV, axis 1 disorders (SCID-I). Individuals were included if they had an active or lifetime major depressive episode, with or without somatic or other psychiatric comorbidities. For this project, only unaffected controls and individuals with an acute or lifetime MDD diagnosis, with or without comorbid anxiety disorder, were included. For a complete description of sample selection, see Supplementary Fig. 1. Upon the completion of baseline participation, individuals were invited to participate in a non-mandatory follow-up examination after 2 years. The study received ethical approval from the Ethics Committees of the Medical Faculties, University of Marburg (07/14), and University of Münster (2014-422-b-S). All participants provided written informed consent allowing the use of their data for the research questions examined in this study, and no additional institutional review board approval was required.

##### Measuring Integrated Novel Dimensions in Neurodevelopmental and Stress-related Mental Disorders (MIND-Set) cohort

The Measuring Integrated Novel Dimensions in Neurodevelopmental and Stress-related Mental Disorders (MINDSet) cohort^22^ was used as a replication cohort. The MIND-Set cohort is an observational, cross-sectional study, which aims to elucidate shared and specific mechanisms of stress-related and neurodevelopmental psychiatric disorders. Individuals aged 18 years and older were recruited through the Department of Psychiatry of the Radboud University Medical Center, Nijmegen, the Netherlands between 2016 and 2021. At the time of recruitment, participants were outpatients at the Department of Psychiatry of the Radboud University Medical Center. The SCID-I for mood and anxiety disorders was conducted by trained clinicians to confirm the MDD diagnosis, alongside other standardized instruments to assess the presence of other psychiatric and neurodevelopmental disorders (see Van Eijndhoven et al.^22^ for a description of all measurement instruments). Individuals were included if they fulfilled the criteria for a stress-related disorder (mood, anxiety or substance use disorders) and/or neurodevelopmental disorder (attention deficit/hyperactivity disorder (ADHD) or autism spectrum disorder (ASD)). For this project, only unaffected controls and individuals with an acute or lifetime MDD diagnosis, with or without comorbid psychiatric disorder(s), were included. For a complete description of sample selection, see Supplementary Fig. 1. A group of individuals free from psychiatric disorders was recruited from the local general population, screened via telephone interviews using the same diagnostic tools. Exclusion criteria comprised active psychotic disorders, IQ estimation <70, sensorimotor disabilities, insufficient Dutch language comprehension, or taking antibiotics at the time of recruitment. The study received ethical approvals from the local medical ethics committee (NL55618.091.015). All participants provided written informed consent allowing the use of their data for the research questions examined in this study, and no additional institutional review board approval was required

From both cohorts, we included participants who provided a fecal sample and had available information regarding MDD diagnosis, medication use, and relevant confounders (section Covariates).

#### Medication use

In the MACS cohort, a trained clinician registered current psychotropic medication use (ATC codes N05* and N06*) through a structured interview. Other medication use was not logged in detail. In the MIND-Set cohort, a pharmacy-employee collected medication history and current use, which was verified by a clinician during the study intake. We grouped psychotropic medications into three categories: SSRIs (ATC codes N06AB*), SNRIs (venlafaxine-N06AX16, milnacipran-N06AX17, duloxetine-N06AX21, vortioxetine-N06AX26), and other psychotropic agents (other N05* and N06*). Supplementary Data 1 provides the full list of psychotropic medications and their corresponding ATC codes.

#### Diagnosis and medication group definitions

**Fig. 1:**
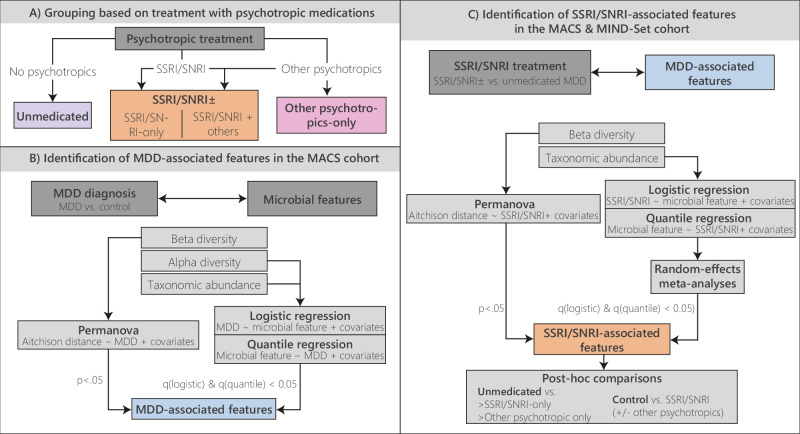
Analysis flowchart. The flowchart depicts the analysis structure. **A** Grouping based on treatment with psychotropic medications into three main groups: unmedicated, SSRI/SNRI-treated and other psychotropics. **B** MDD-associated microbial features were identified in the MACS cohort. All models were adjusted for age, gender, BMI, sequencing depth, sequencing batch, and collection site. **C** All MDD-associated microbial features were tested for associations with SSRI/SNRI treatment in the MACS and MIND-Set cohorts. These models were also adjusted for psychiatric comorbidities and depression symptom severity. MDD Major Depressive Disorder, FDR False Discovery Rate, SSRI Selective Serotonin Reuptake Inhibitor, SNRI Serotonin-Norepinephrine Reuptake Inhibitor.

We grouped participants based on the MDD diagnosis: individuals with an MDD diagnosis (active or a lifetime episode) were assigned to the MDD group. Individuals without any psychiatric diagnoses were assigned to the unaffected control group. Within the patient group, we stratified patients into three groups based on their use of SSRIs/SNRIs and other psychotropic medication (Fig. 1A). Patients not treated with any psychotropic medication were assigned to the unmedicated group. Patients treated with at least one SSRI or SNRI, either with or without co-medication with other psychotropic medications, were assigned to the SSRI/SNRI± group. Patients treated with only other psychotropic medication (no SSRI/SNRI) were assigned to the other psychotropic-only group. Non-psychotropic medications were not considered for this grouping. We focused on SSRIs and SNRIs, the most commonly prescribed pharmacotherapies for MDD, which primarily act by targeting the serotonin transporter (SERT). This choice enhances mechanistic interpretability because SERT is highly expressed in the gastrointestinal tract, where it modulates gut motility^23,24^, and influences the microbial community^25^.

### Microbiota data acquisition and preprocessing

The protocol for DNA extraction, sequencing and preprocessing has been described in detail elsewhere for MACS^26^ and MIND-Set^27^. In short, participants from the MACS cohort were asked to collect a fecal sample on the day of their baseline intake interview or within two weeks after. Baseline samples were used by default; if unavailable, the 2-year follow-up samples were used along with corresponding clinical and questionnaire data. The DNA was extracted from the fecal samples, and the V1-V2 regions of the 16 s rRNA gene were amplified using the 27 F − 338 R primer pair (F:AGAGTTTGATCMTGGCTCAG, R:GCTGCCTCCCGTAGGAGT). Sequencing was performed using the Illumina MiSeq v3 platform (2x300bp), carried out in two batches, with samples from different timepoints processed in separate batches. Sequencing yielded an average of 36,335 [4008–11,3711] paired-end reads per sample. Raw sequences were pre-processed using the Natrix pipeline^28^, which clustered the reads into OTUs before using the BLAST algorithm against the SILVA database for taxonomic assignment^29^. The resulting feature table contained a total of 48,495 OTUs, corresponding to 636 genera.

For the MIND-Set cohort, all participants collected a fecal sample at home. DNA was extracted from the fecal samples, and the V4 region of the 16s rRNA gene was amplified using the 515F-806R primer pair (F:GTGCCAGCMGCCGCGGTAA, R:GGACTACHVGGGTWTCTAAT). Sequencing was performed using the Illumina NovaSeq6000 platform (2x250bp) in one batch, yielding an average of 768,466 [226,130 –1,266,720] paired-end reads per sample. Raw sequences were pre-processed using the QIIME2-DADA2 pipeline^30,31^, which inferred ASVs and then assigned taxonomy using a Naïve Bayes classifier pre-trained on the SILVA v138 reference database. The resulting feature table contained a total of 21,078 ASVs, corresponding to 243 genera.

### Microbial diversity & taxonomy

All analyses were performed in R (version 4.4.1^32^), using the *mia*, *microbiome*, *vegan*, and *phyloseq* packages^33–35^. Alpha diversity, reflecting within-sample richness and evenness, was quantified using the Inverse Simpson and Pielou’s indices^36^. These indices were selected as they showed the lowest correlation with other indices, indicating complementary information. Beta diversity, representing global between-sample differences in microbial composition, was quantified using pairwise Aitchison distances derived from center log-ratio (CLR) transformed abundance data^37^.

For taxonomic analyses, the microbial feature table was aggregated to the genus level. Genera detected in less than 10% of all the samples were excluded, which has shown to improve reproducibility across studies^38^. The remaining genera were centered log-ratio (CLR)-transformed to account for zero inflation and compositionality in the data^39^.

### Statistics and reproducibility

To investigate the extent to which microbial differences in MDD are modified by SSRI/SNRI treatment, we conducted a multi-step analysis. First, we identified microbial associations (alpha/beta diversity, taxonomy) with MDD diagnosis in the larger, well-powered MACS cohort (*N* = 1568; Fig. 1B). Next, both in the MACS and MIND-Set (*N* = 234) cohorts, we assessed whether MDD-associated genera were also associated with SSRI/SNRI± treatment (Fig. 1B). We then performed a meta-analysis of the association between the gut microbiome and SSRI/SNRI treatment across the two cohorts. Finally, we performed post-hoc analyses to examine whether these associations were specifically driven by SSRI/SNRI treatment.

#### Microbial diversity and taxonomy

##### Associations with MDD

Associations between the microbiome and MDD were tested in MACS. For alpha diversity, we applied logistic regression (MDD diagnosis as the outcome, alpha diversity as the predictor) and quantile regression (median alpha diversity as the outcome, MDD diagnosis as the predictor). Logistic regression is appropriate for binary outcomes and captures non-linear associations^40^. Quantile regression makes minimal distributional assumptions and is robust to outliers, making it well-suited for sparse microbiome data^41^. Beta diversity differences between MDD groups were assessed with PERMANOVA using the Aitchison distance matrix as the outcome and MDD diagnosis as the predictor. To identify MDD-associated genera, we applied the same logistic and quantile regression models as for alpha diversity, fitting one model per genus. All models were adjusted for covariates (section *Covariates*), and *p*-values were adjusted for false discovery rate (FDR) using the Benjamini-Hochberg procedure, with significance set at p_FDR_ < 0.05.

##### Associations with SSRI/SNRI treatment

Associations between the microbiome and SSRI/SNRI treatment were tested in both MACS and MIND-Set. For all diversity indices and microbial genera significantly associated with MDD, we repeated the same analysis scheme within the (stratified) MDD group, contrasting the SSRI/SNRI± against the unmedicated group. Finally, to improve statistical power, we performed a random-effects meta-analysis across both cohorts, combining the effect sizes from logistic and quantile regression assessing the association between SSRI/SNRI± treatment and the MDD-associated genera. Effect-sizes were weighted by within-study variance and between-study heterogeneity^42^.

#### SSRI/SNRI specificity

Two post-hoc analyses were performed: first, to test whether associations were specific to SSRI/SNRI treatment, we compared SSRI/SNRI-only and other-psychotropic-only groups against unmedicated patients. Second, to determine whether case-control differences were influenced by medication, we compared the SSRI/SNRI± and unmedicated MDD groups with unaffected controls. Both quantile and logistic regression models were assessed (Fig. 1C).

#### Covariates

All statistical models were adjusted for age, sex, and BMI as covariates, given their known associations with gut microbiome composition and/or MDD^12,43^. Sequencing depth (the number of reads retained after pre-processing) was included in all models, as it was significantly associated with the number of detected microbial features in both the MACS (*r* = .23, *p* < .001) and MIND-Set (*r* = .09, *p* < .001) cohorts. In the MACS cohort, sequencing batch was associated with the number of detected OTUs (*F* = 36.92, *p* < .001; Supplementary Fig. 2) and was therefore included as an additional covariate in all models.

Models examining associations with SSRI/SNRI treatment were further adjusted for depression symptom severity, assessed with the BDI-II^44^ in MACS and the IDS-SR^45^ in MIND-Set, as severity differed significantly between medication groups in both cohorts (MACS: *F* = 61.32, *p* < .001; MIND-Set: *F* = 14.68, *p* < .001). These models were also adjusted for the number of psychiatric comorbidities. Supplementary Data 2 provides a graphic overview of covariate adjustment across models.

#### Sensitivity analyses

To assess the robustness of associations between SSRI/SNRI treatment and gut microbiota composition in MDD, sensitivity analyses were performed to control for remission status, appetite change, somatic comorbidity and three dietary factors (Supplementary Data 2). In both cohorts, we examined whether associations persisted after accounting for remission status, categorized as ‘active’, ‘partially remitted’, and ‘remitted’ in MACS, and as ‘active’ and ‘remitted’ in MIND-Set. In the MACS cohort, additional sensitivity analyses adjusted for appetite change, somatic comorbidity, and dietary factors. Appetite change was assessed using item A3 from the SIGH-HAMD^46^ (*“Has your appetite been greater than when you feel well or okay?”*). Somatic comorbidity was encoded as a binary variable reflecting the presence or absence of any somatic diagnosis. Dietary fiber intake (g), total calorie intake (kJ), and soft drink consumption¹⁴ were derived from a validated German semiquantitative food frequency questionnaire (FFQ2)^47^. Sensitivity analyses were applied to all microbial features significantly linked to SSRI/SNRI use, using logistic and quantile regression models, with the sensitivity variable included as an additional covariate.

## Results

### Cohort characteristics

**Fig. 2:**
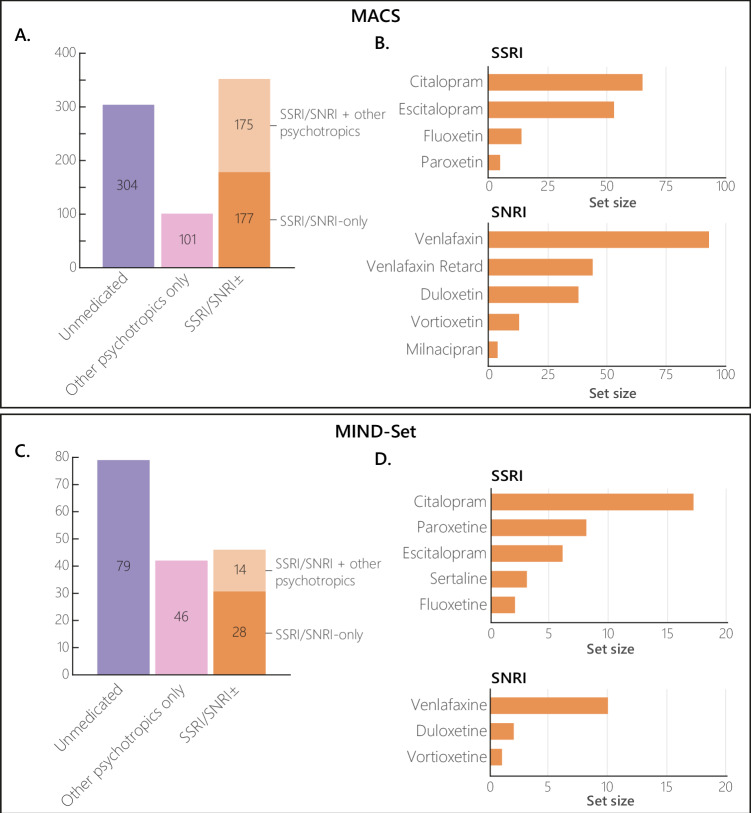
SSRI and SNRI treatment. An overview of the distribution of medication groups in the MACS (**A**) and MIND-Set (**C**) cohorts, along with the individual SSRI and SNRI types within the MACS (**B**) and MIND-Set (**D**) cohorts.

Table 1 and Supplementary Data 3 provide a demographic, clinical and technical overview of the MACS and MIND-Set cohorts. The MACS cohort included 811 unaffected controls and 757 MDD patients. Among MDD patients, 352 were treated with SSRIs/SNRIs (*n* = 177 SSRI/SNRI-only; *n* = 175 SSRI/SNRI + other psychotropics), 101 used only other psychotropics, and 304 were unmedicated. Four unaffected controls using psychotropic medications were excluded. The MIND-Set cohort comprised 67 controls and 167 MDD patients. Among MDD patients, 42 were treated with SSRIs/SNRIs (*n* = 28 SSNRI/SNRI-only and *n* = 14  SSRI/SNRI + other psychotropics), 46 used only other psychotropics, and 79 were unmedicated. An overview of individual SSRI/SNRI types used in MACS and MIND-Set is presented in Fig. 2 and Supplementary Fig. 3.

**Table 1:** Demographic characteristics of the MACS and MIND-set cohorts

			*N*	Age (mean (SD))	Sex (M/F)	BMI (mean (SD))	Depression symptom severity^a^	Remission status (active episode/partial remission/remission)	# psychiatric comorbidities (1/2/3/4 + )
MACS	Unaffected control		811	35.7 (13.3)	282/529	24.4 (4.66)	3.86 (4.2)	-	-
	MDD	All	757	37.1 (13.0)	257/500	26.2 (5.88)	16.9 (11.3)	294/187/275	585/172/-/-
		Unmedicated	304	35.6 (12.6)	89/216	25.6 (6.01)	13.1 (9.67)	74/57/172	246/58/-/-
		SSRI/SNRI ±	352	38.1 (13.0)	122/230	26.7 (5.95)	19.6 (11.4)	180/91/81	257/95/-/-
		Other psychotropics only	101	38.6 (14.0)	46/55	26.4 (5.08)	19.4 (12.4)	40/39/22	82/19/-/-
MIND-Set	Unaffected control		67	39.0 (16.6)	33/0/34	23.9 (4.4)	5.15 (4.5)	-	-
	MDD	All	167	42.0 (14.7)	87/0/80	26.6 (10.2)	34.2 (13.0)	92/0/75	30/51/34/52
		Unmedicated	79	36.7 (13.5)	45/0/34	24.6 (4.7)	29.7 (11.9)	35/0/44	11/30/13/25
		SSRI/SNRI±	42	49.9 (13.7)	19/0/23	28.2 (4.9)	38.9 (13.6)	29/0/13	11/13/8/10
		Other psychotropics only	46	44.3 (14.1)	23/0/23	28.6 (17.6)	37.8 (11.7)	28/0/18	8/8/13/17

^a^Depression symptom severity is measured using BDI in MACS and using the IDS-SR in MIND-Set.

*MDD* Major Depressive Disorder, *SSRI/SNRI* ±  SSRI/SNRI +/- other psychotropic medication.

In both cohorts, MDD patients were older and had a higher BMI than controls. The SSRI/SNRI group was also older, with a higher BMI and depression severity than the unmedicated group. In MACS, the SSRI/SNRI group had a higher proportion of males than the unmedicated group (35% vs. 29%) (Supplementary Data 4).

### Microbial associations with MDD

We found no significant associations between MDD diagnosis and alpha diversity (Supplementary Data 5), but there was a significant association between Aitchison’s distance and MDD diagnosis, *F* = 1.660, *R*^2^ = 0.001, *p* = 0.011 (Supplementary Data 6). The full regression models, including covariate effects, are presented in Supplementary Data 16–31.

Nine out of 188 genera showed a statistically significant association with MDD diagnosis in both logistic and quantile regression models (Supplementary Fig. 4 and 5, Supplementary Data 7–9). Confirming previous findings in this cohort^26^, seven genera showed a higher abundance in individuals with MDD, including *Hungatella* and *Eggerthella*, while the remaining two, *Clostridium sensu stricto 1* and *Lachnospiraceae FCS020 group*, showed a lower abundance. An additional eight genera were identified only through logistic regression, and five genera were only identified through quantile regression (Supplementary Data 8 and 9).

### Microbial associations with SSRI/SNRI treatment

#### MACS

**Fig. 3:**
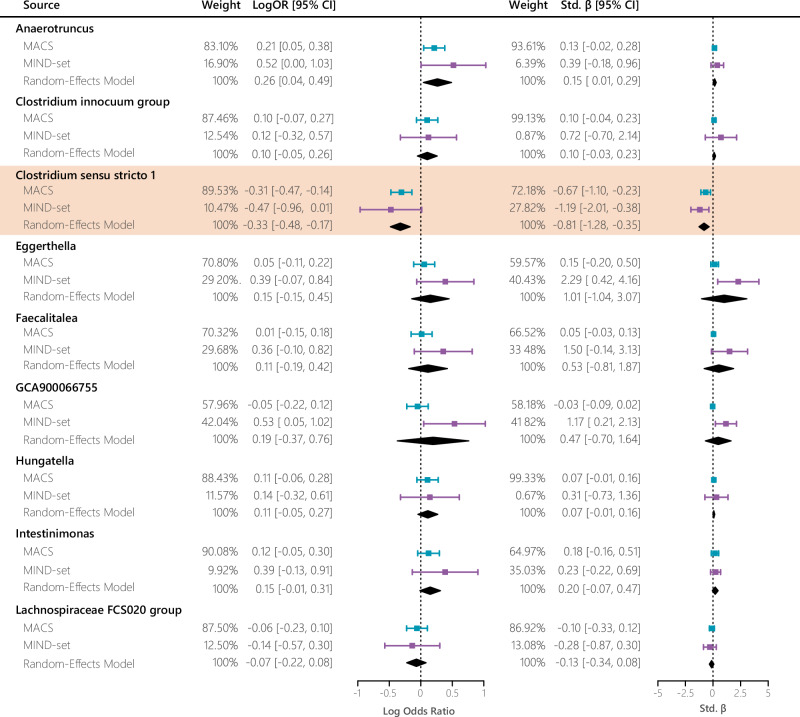
Meta-analysis forest plot of the associations between genera and SSRI/SNRI treatment. Outcomes of the random-effects meta-analysis associations between SSRI/SNRI treatment and MDD-associated genera from logistic regression (left) and quantile regression (right). The percentages represent the study weight. The squares represent log odds ratios (logistic regression) and beta coefficients (quantile regression). The confidence bars represent the study-level 95% confidence interval. The diamond width represents the confidence interval (95% CI) of the (overall) summary estimate.

In the MACS cohort, there were no significant differences in Aitchison’s distance between the SSRI/SNRI± and unmedicated patient groups, F = 1.078, R^2^ = .002, *p* = .232 (Supplementary Data 6). Out of the nine MDD-associated genera, one was also associated with SSRI/SNRI± treatment: *Clostridium sensu stricto 1* showed a significantly lower abundance in SSRI/SNRI ± -treated compared to unmedicated patients, both in the logistic and quantile regression (Fig. 3, Supplementary Data 10). None of the other covariates (age, sex, BMI, anxiety symptoms, depression symptoms, collection site and sequencing depth,) were significantly associated with *Clostridium sensu stricto 1* abundance in the quantile regression model (Supplementary Data 18).

#### MIND-Set

In the MIND-Set cohort, there were no significant differences in Aitchison’s distance between the SSRI/SNRI± and unmedicated patient groups, *F* = 1.029, *R*^2^ = 0.012, *p* = 0.306 (Supplementary Data 6). Taxonomic analyses revealed a significantly lower abundance of *Clostridium sensu stricto 1* in SSRI/SNRI-treated compared to unmedicated patients in the quantile regression analysis (Fig. 3). In logistic regression analysis the association was only nominally significant (Supplementary Data 10).

#### Meta-analysis

Random-effects meta-analysis showed a significant negative association between SSRI/SNRI± treatment and *Clostridium sensu stricto* 1, consistent across logistic and quantile regression analyses (Fig. 3). *Anaerotruncus* showed a nominal positive association with SSRI/SNRI± treatment in both quantile and logistic regression meta-analyses, although this did not survive correction for multiple testing and was not consistently observed in the individual cohorts. No associations with SSRI/SNRI± treatment were detected for the other seven MDD-associated genera (Supplementary Data 11).

#### SSRI/SNRI specificity

The identified associations with *Clostridium sensu stricto 1* abundance appear to be specific to the use of SSRI/SNRIs. Across both cohorts, *Clostridium sensu stricto 1* abundance was significantly lower in the SSRI/SNRI-only group but showed no differences in the other-psychotropic-only group, compared to unmedicated patients (Supplementary Data 12).

Moreover, the reduced *Clostridium sensu stricto 1* abundance observed in MDD patients compared to controls also seemed to be driven by SSRI/SNRI treatment. When comparing the SSRI/SNRI± and the unmedicated MDD groups to unaffected controls, stronger associations were found in the SSRI/SNRI± group for most MDD-associated genera (Supplementary Fig. 5, Supplementary Data 13). Specifically, across both cohorts, the association with *Clostridium sensu stricto 1* was significant and exhibited larger effect sizes in the SSRI/SNRI± group, whereas no significant association was observed, and effect sized were markedly smaller, in the unmedicated group (Supplementary Fig. 5, Supplementary Data 13).

#### Sensitivity analyses

The association between *Clostridium sensu stricto 1* and SSRI/SNRI treatment remained consistent after adjustment for remission status, appetite changes, and somatic comorbidities. Dietary adjustment (fiber intake, caloric intake, and soft drink consumption) was possible in a subset of participants from the MACS cohort where these data were available (N = 222). Effect sizes remained comparable to the unadjusted models (Supplementary Data 14), suggesting that dietary differences between treatment groups do not explain the observed reduction in *Clostridium sensu stricto 1*. Associations were, however, no longer statistically significant, which is likely attributable to the reduced sample size rather than confounding by diet.

## Discussion

In this study, we aimed to identify the extent to which SSRIs and SNRIs modify gut microbial differences associated with MDD, thereby distinguishing disorder-related signals from those more likely shaped by pharmacological treatment. We identified nine genera with differential abundance between MDD patients and controls, including elevated *Eggerthella* and *Hungatella*. This replicates findings in a largely overlapping sub-sample of the MACS cohort^26,48^ and from independent studies^4,27,49^, especially the independently replicated findings underscoring their consistency as markers of the disorder. Among these taxa, *Clostridium sensu stricto 1* showed a distinct pattern in relation to medication: its reduced abundance in MDD patients appeared to be driven by SSRI/SNRI treatment, with lower levels observed in treated compared to unmedicated patients, and its association with MDD diagnosis only evident in the treated group. This identifies *Clostridium sensu stricto 1* as a potentially medication-sensitive genus, while the other MDD associated genera, which were not affected by medication status, may reflect other disorder-related factors instead, such as host genetics of lifestyle. *Anaerotruncus* showed only nominal associations in the meta-analysis across cohorts, suggesting caution in interpretation. This association does align with a previously reported association between *Anaerotruncus colihominis* and psychotropic medications use^16^. Therefore, further research into *Anaerotruncus*, particularly regarding medication specificity and the functional implications of its increased abundance, is warranted.

Clinical studies examining the impact of SSRIs and SNRIs on the gut microbiome show mixed results: some cross-sectional studies show decreased *Clostridium* or *Clostridiaceae* abundance with SSRI/SNRI treatment^10,13,50^, whereas others find no such differences but report associations with other taxa^16,17,51^. These inconsistencies may result from small sample sizes, and incomplete adjustment for clinical and demographic factors like symptom severity, psychiatric comorbidities, socioeconomic status, and geographical location. Since medicated and unmedicated patients often differ in these factors, insufficient control can obscure antidepressants’ effects on gut microbiota. Our study overcomes this by stratifying patients by medication status and adjusting for demographic and clinical covariates, allowing more precise microbiome-antidepressant association estimates.

Mechanistically, SSRIs and SNRIs act by inhibiting the serotonin transporter (SERT) in the central nervous system, but they also exert unintentional effects in the gut that may directly or indirectly affect the microbiome. For example, SSRIs and SNRIs can influence gut motility via serotonin receptor modulation, potentially causing diarrhea or constipation and indirectly altering the microbiome^52^. In addition, these antidepressants have direct antimicrobial effects *in vitro*: they can inhibit bacterial growth by penetrating bacterial membranes, disrupting efflux pump function, and compromising membrane integrity^53–55^. This may particularly affect Gram-positive bacteria, including *Clostridium sensu stricto 1*, due to their membrane structure and limited efflux capacity in comparison to Gram-negative bacteria^56^. Supporting this, metagenomic analyses in clinical cohorts revealed that the SSRI escitalopram reduced the abundance of *Clostridium* species and induced upregulation of bacterial survival pathways, including efflux pumps, antibiotic resistance, and sporulation genes, paralleling experimental findings^15^. These antimicrobial actions may impact host-microbe interactions, including peripheral serotonin metabolism. That is, Gram positive spore-forming bacteria (such as *Clostridium sensu stricto 1*) regulate gut-derived serotonin by producing metabolites that stimulate serotonin synthesis by the enterochromaffin cells^57,58^. Reductions in the abundance of these bacteria during SSRI/SNRI treatment may therefore alter gut serotonin dynamics, as shown preclinically for the spore-forming *Turicibacter sanguinis*^57^. Consistently, clinical studies have shown that SSRI/SNRI treatment induced decreases in spore-forming bacteria alongside shifts in peripheral serotonin metabolism^15,59^. The findings from this study depend on 16S-derived taxonomic profiles, limiting the ability to draw conclusions about functional changes. Establishing the functional relevance of these findings will require complementary approaches, including experimental validation and/or metagenomic, metatranscriptomic, or proteomic analyses.

Emerging evidence furthermore links SSRI-induced shifts in microbial composition and metabolic activity to treatment outcomes, including lower abundance of sporulation genes and higher plasma tryptophan in SSRI responders^14,15,59^. Notably, an open-label trial in treatment-resistant depression found that adjunctive administration of *Clostridium butyricum* (a member of the *Clostridium sensu stricto 1* genus) improved treatment response^60^, suggesting that abundance of this species, whether pre-existing or medication-induced, may modulate treatment efficacy. Prospective studies incorporating repeated multi-omics sampling and detailed clinical phenotyping are needed to establish whether these associations are causal. This will guide the development and implementation of adjunctive interventions, such as targeted probiotics (e.g., *C. butyricum)* or dietary strategies, to improve therapeutic outcomes.

Our study benefits from a large, well-characterized patient cohort and independent replication, strengthening confidence in and generalizability of the findings. Nonetheless, several limitations remain. First, limited data on medication dose, treatment duration, and follow-up constrained our ability to distinguish acute from chronic medication effects and to link microbial changes to symptom trajectories. Our findings likely reflect long-term microbial adaptations rather than short-term effects, which may involve different mechanisms or temporal dynamics. Second, limited information on side effects, medication dosage, comedication and treatment duration, restricted our ability to assess the effect of medication burden. However, given that observed associations were not replicated in patients treated with non-SSRI/SNRI psychotropics, it seems unlikely that the signal is explained by general medication burden alone. Third, while evidence shows that microbial susceptibility varies not only by antidepressant class, but also by the specific compound^7,9,11^, subgroups of individual antidepressants were generally small and highly imbalanced, precluding assessment of compound-level effects on the gut microbiome. Still, we could distinguish that the observed associations are specific to SSRIs/SNRIs and do not generalize to other classes of psychotropic medication. Finally, reliance on fecal samples limits insight into the small intestine (and its microbiome), where SSRIs and SNRIs are thought to exert their primary effects due to higher expression of the serotonin transporter (SERT)^61^. As a result, drug-microbiome interactions occurring in upper intestinal sites may not be captured.

## Conclusion

This study shows that SSRI/SNRI treatment shapes gut microbial alterations in MDD, with reductions in *Clostridium sensu stricto 1* driven by antidepressant use, suggesting a role for gut serotonin metabolism. These findings clarify the drivers of microbial alterations in depression and lay the groundwork for microbiome-targeted interventions to improve treatment outcomes in MDD.

## Supplementary information

Transparent Peer Review file

Supplemental Information

Description of Additional Supplementary Files

Supplementary Data 1-31

## Data Availability

The code supporting the findings of this study, along with software versions, is available at 10.5281/zenodo.20394496.

## Data availability

The raw clinical metadata, and microbial community datasets generated and analyzed during this study are available from Tim Hahn (hahnt@uni-muenster.de) upon reasonable request. Anonymized data can be accessed directly, provided that the intended use of the data is clearly specified in writing at the time of request. Requests will be reviewed within a timeframe of up to four weeks. All summary statistics produced in this study are included with the manuscript as Supplementary Data 16–3^1^. The source data for Fig. 2 are provided in Supplementary Data 14, and the source data for Fig. 3 are provided in Supplementary Data 11.
