# Supplementary material for: The effect of SSRI/SNRI antidepressant treatment on the gut microbiota of patients with major depressive disorder
Source: Commun Med (Lond). 2026 Jul 16;6:399. doi: 10.1038/s43856-026-01782-5 (PMC13376374; doi:10.1038/s43856-026-01782-5)

The effect of SSRI/SNRI antidepressant treatment on the gut microbiota of patients with major depressive disorder

Eugenia Emile Natasha, MSc[1,16]*, Danique Mulder MSc[1]*, Leon Fehse MSc[2], Nils R. Winter PhD[3], Lukas Fisch MSc[3], Marius Welzel PhD[2], Corinna Bang PhD[4], Susanne Meinert MSc[3,5], Kira Flinkenflügel MSc[3], Tiana Borgers PhD[3], Janik Goltermann PhD[3], Elisabeth J. Leehr PhD[3], Carsten Culmsee PhD[6,7], Frederike Stein PhD[8], Florian Thomas-Odenthal PhD[8], Paula Usemann MSc[8], Lea Teutenberg MSc[8], Igor Nenadic PhD[8], Benjamin Straube PhD[8], Nina Alexander PhD[8], Hamidreza Jamalabadi PhD[8], Andreas Jansen PhD[8], Robert Nitsch MD PhD[5], Andreas Lügering MD[9], Andre Franke PhD[4], Udo Dannlowski MD, PhD[3,10], Tilo Kircher MD[6,8], Dominik Heider PhD[2], Tim Hahn PhD[3], Janna N. Vrijsen PhD[1,11,12], Philip van Eijndhoven MD PhD[1], Indira Tendolkar MD PhD[1], Andreas Reif MD[13,14], Sharmili Edwin Thanarajah MD[13,14,15], Silke Matura PhD[13], Alejandro Arias Vasquez PhD[1]#, Mirjam Bloemendaal PhD[13,15]#^

Supplementary Figures

Supplementary Figure 1. Flowchart for participant selection 2

Supplementary Figure 2. Sequencing batch effect in the MACS cohort.. 3

Supplementary Figure 3. Distribution of treatment psychotropic medications 4

Supplementary Figure 4. Abundance distribution MDD-associated genera 5

Supplementary Figure 5. Associations between genus abundance and MDD and SSRI/SNRI treatment.. 7

Supplementary Figure 1. Flowchart for participant selection

Summary of the number of participants included/excluded in every step in the **(A)** MACS and **(B)** MIND-Set cohort. We selected participants based on several criteria: available fecal sample; MDD diagnosis for patients; available data about age, gender, BMI, and symptom severity; and not on any psychotropic medication for the unaffected controls

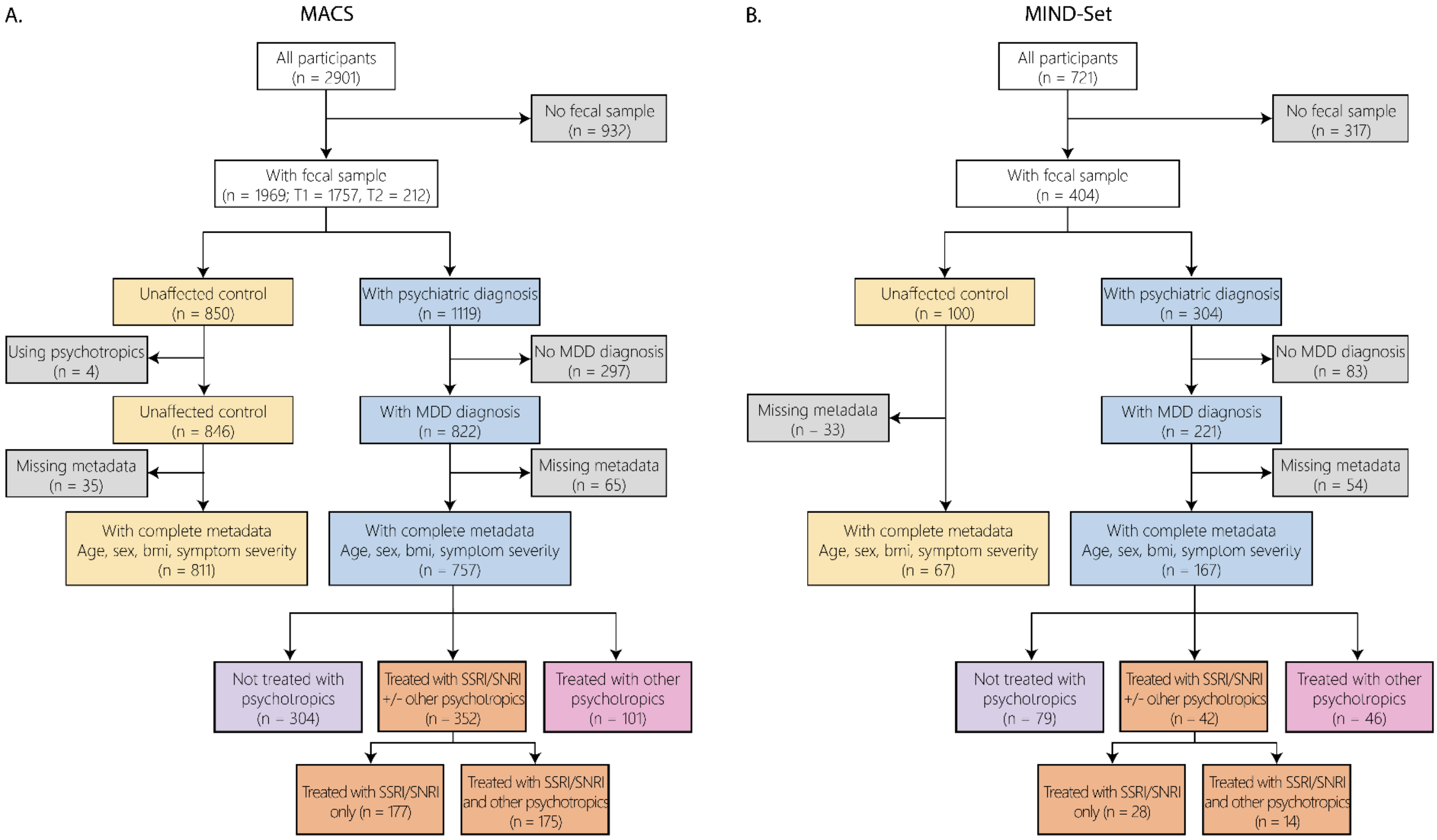

Supplementary Figure 2. Sequencing batch effect in the MACS cohort

In the MACS cohort, unsupervised ordination (PCA on Aitchison distance) on OTU-level revealed a sequencing batch effect **(A)**. After agglomeration to genus level, the sequencing batch effect disappeared **(B)**. Since the batch affected the microbial community structure, we corrected for batch effect in all statistical models.

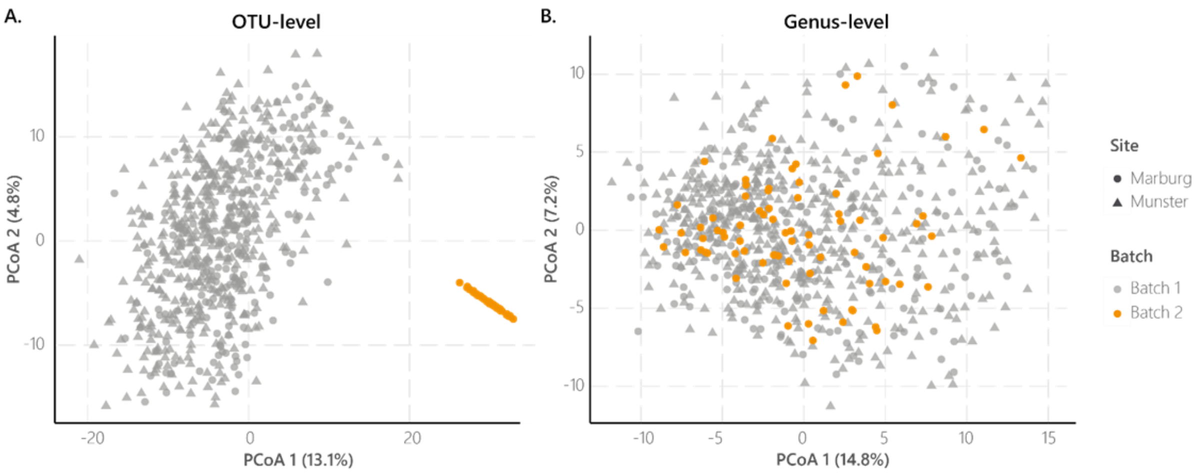

Supplementary Figure 3. Distribution of treatment psychotropic medications

The distribution of medication use in the MACS **(A)** and MIND-Set **(C)** cohort together with corresponding groups: unmedicated in purple, SSRI/SRNRI (+/- other psychotropics) in orange and other psychotropics only in pink. The horizontal bars show the number of participants using each corresponding medication. The dots represent the combination of different types of medications. The vertical bars show the number of participants treated with this combination of medications. Panel **(B)** and **(D)** show the different medications non-SSRI/SNRI psychotropic medications for MACS and MIND-Set, respectively.

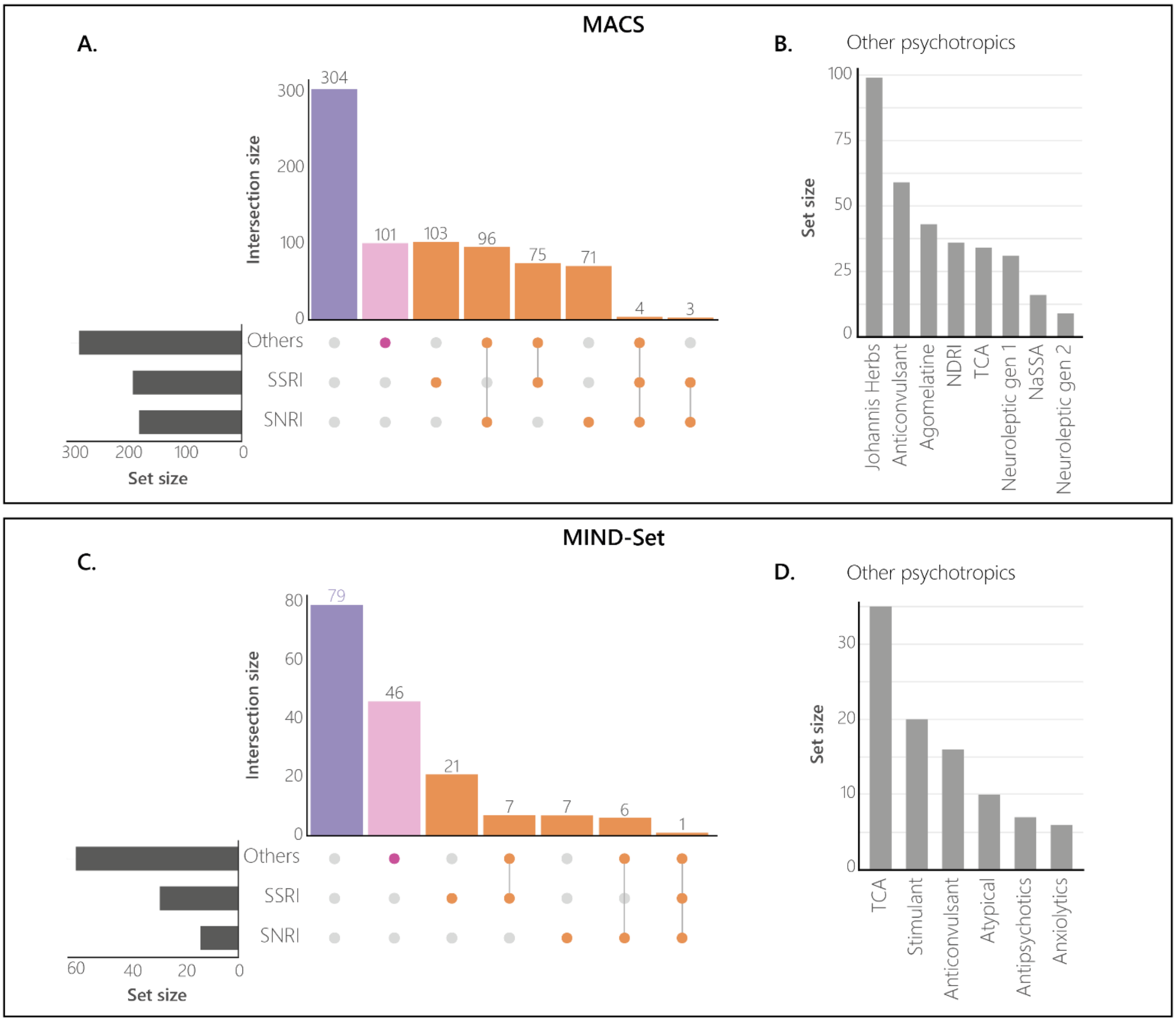

Supplementary Figure 4. Abundance distribution MDD-associated genera

Density plots of centered log-ratio-transformed (CLR) abundance of the nine MDD-associated bacterial genera for the unaffected controls (yellow), MDD (blue), SSRI/SNRI-treated MDD (orange) and unmedicated MDD (purple) groups in the MACS cohort (left) and MINDSet cohort (right)

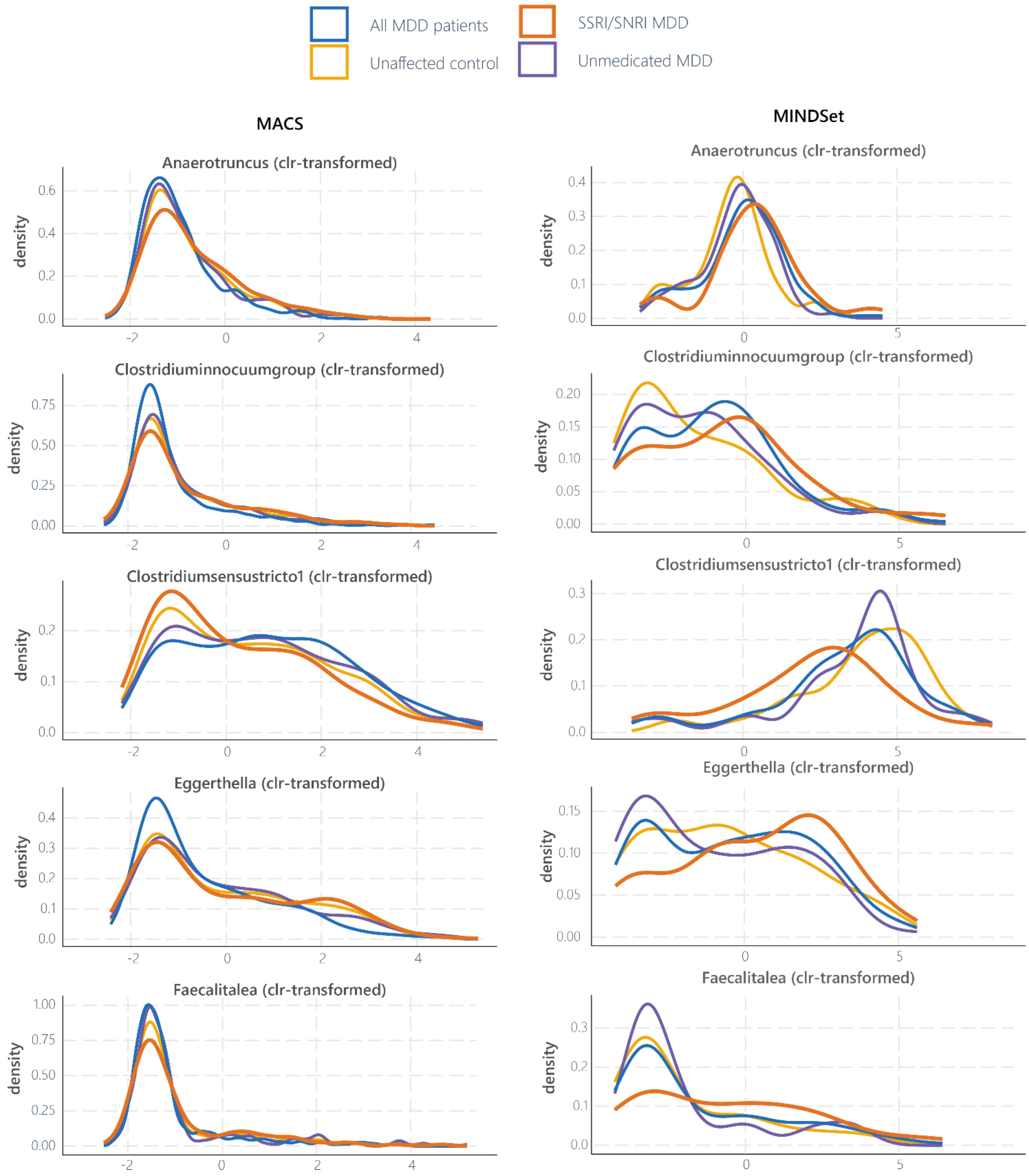

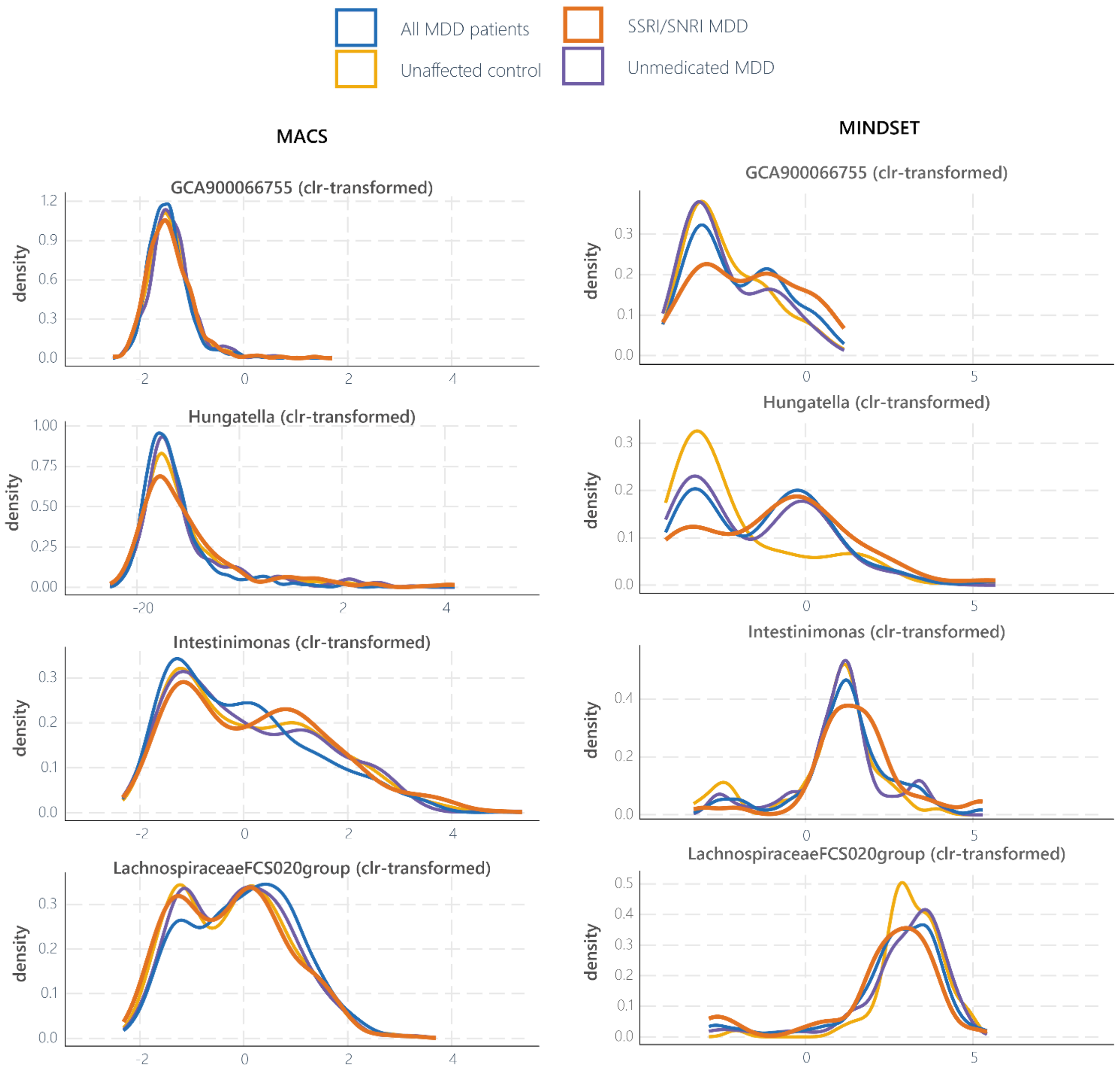

All MDD patients
Unaffected control
SSRI/SNRI MDD
Unmedicated MDD
MACS
MINDSET
GCA900066755 (clr-transformed)
Hungatella (clr-transformed)
Intestinimonas (clr-transformed)
LachnospiraceaeFCS020group (clr-transformed)
density

Supplementary Figure 5. Associations between genus abundance and MDD and SSRI/SNRI treatment

Heatmaps showing the beta coefficients (quantile regression, left) or logOR (logistic regression, right) of the group differences between the MDD (all, SSRI/SNRI, unmedicated) and unaffected control groups, and of the group differences between the SSRI/SNRI-treated and the unmedicated MDD groups. Higher beta coefficients/logOR (red) indicates a higher abundance in the first group of each comparison (i.e, the MDD groups in the case-control and the SSRI/SNRI group in the medication comparison).

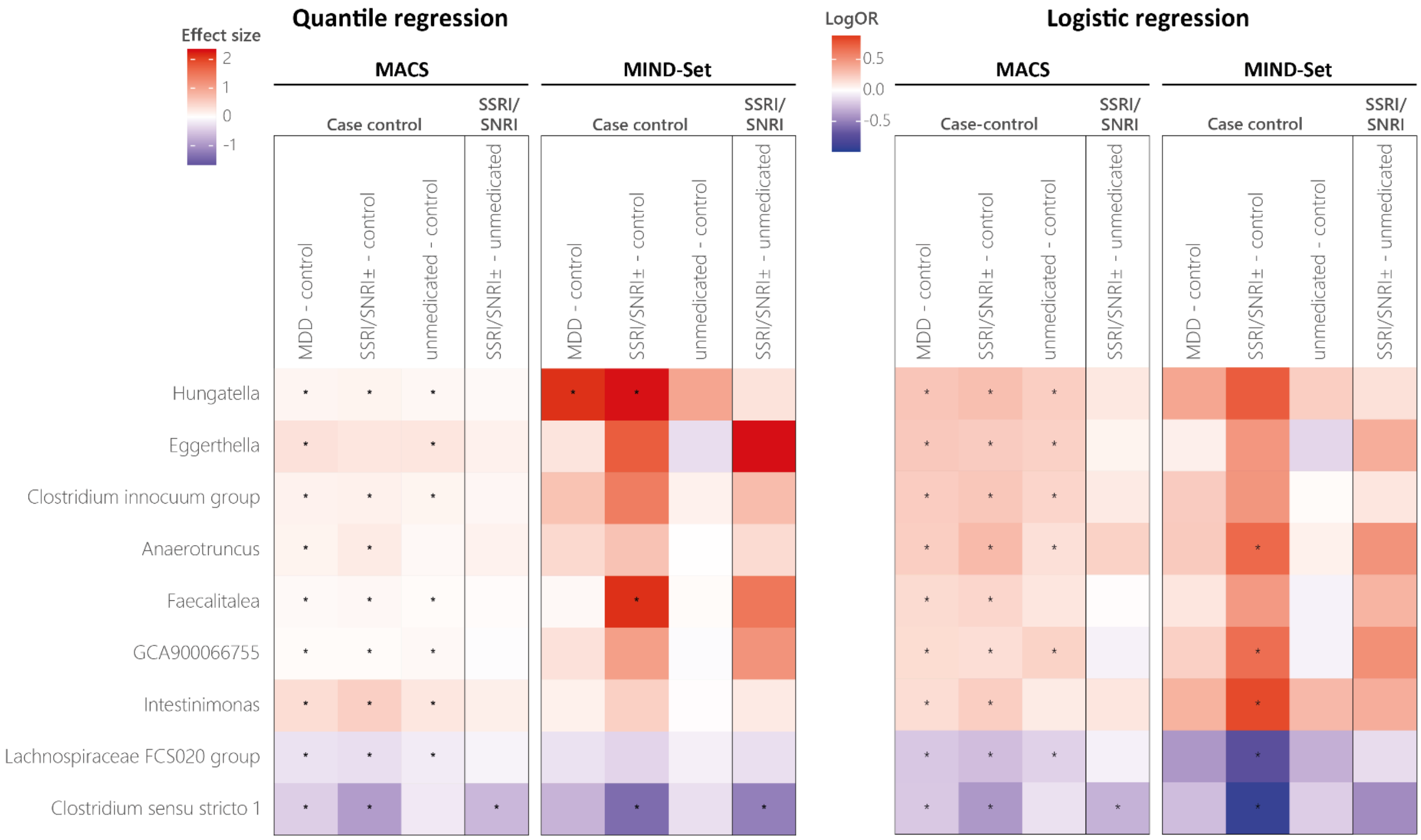

Supplement: Supplementary file 2 — Supplemental Information [file 43856_2026_1782_MOESM2_ESM.pdf]
