# Supplementary material for: The effect of SSRI/SNRI antidepressant treatment on the gut microbiota of patients with major depressive disorder
Source: Commun Med (Lond). 2026 Jul 16;6:399. doi: 10.1038/s43856-026-01782-5 (PMC13376374; doi:10.1038/s43856-026-01782-5)
Supplement: Supplementary file 3 — Description of Additional Supplementary Files [file 43856_2026_1782_MOESM3_ESM.pdf]

Supplementary Data Information File

The effect of SSRI/SNRI antidepressant treatment on the gut microbiota of patients with major depressive disorder

Natasha, Mulder et al.

An overview of the 31 supplementary data files accompanying the manuscript. The first 15 tables contain the main analysis results (microbial associations with MDD diagnosis and SSRI/SNRI treatment), including demographic characteristics of the MACS and MINDSet cohorts, covariate correlations, diversity analyses, genus-level association statistics, meta-analytic effect sizes, and medication subgroup analyses. Tables 16–31 contain the full summary statistics of those analyses including the results of the covariates in the models.

Data Table	Sheet name	Description
**1**	**ATC Codes**	An overview of the medication classes used in the MACS and MINDSet cohorts, along with their corresponding ATC codes and ATC classification
**2**	**Covariates**	An overview of covariate adjustment across statistical models
**3**	**Demographic table**	Demographic, clinical and technical characteristics of the MACS and MIND-Set cohorts
**4**	**Cov. Correlations**	Overview of the correlations between MDD diagnosis, SSRI/SNRI treatment and covariates.
**5**	**Alpha diversity (MACS)**	Overview of the associations between MDD diagnosis (case-control), SSRI/SNRI treatment and alpha diversity.
**6**	**Beta diversity**	Overview of the associations between MDD diagnosis (case-control), SSRI/SNRI treatment and beta diversity.
**7**	**Prevalence**	The prevalence (% samples with count >0) of the 9 MDD-associated genera in unaffected controls, MDD patients and medication groups for MACS and MIND-Set
**8**	**MACS case-control**	Overview of the associations between MDD diagnosis (case-control) and genus abundance in the MACS cohort
**9**	**MINDSet case-control**	Overview of the associations between MDD diagnosis (case-control) and genus abundance in the MINDSet cohort
**10**	**Medication statistics**	Overview of the associations between SSRI/SNRI treatment and genus abundance in the 9 MDD-associated genera
**11**	**Meta-analysis**	Overview of the pooled effect sizes from the random-effects meta-analysis of the associations between SSRI/SNRI treatment and genus abundance in the MACS and MIND-Set cohorts
**12**	**SSRI SNRI specificity**	Subgroup analyses in SSRI/SNRI-only and other psychotropic-only medication groups
**13**	**Comparison to control**	Subgroup analyses comparing the unmedicated and SSRI/SNRI-treated MDD groups to unaffected controls.
**14**	**Sensitivity analyses**	Sensitivity analyses assessing the robustness of the statistical models for remission, appetite change, somatic comorbidity and dietary factors
**15**	**Medication Groups**	Medication group sample sizes in the MACS and MINDSet cohorts
**16**	**Alpha covariates**	Full regression models for alpha diversity associations in MACS, corresponding to Supplementary Table 5, including all covariates.
**17**	**MACS case-control cov**	Full regression models for MDD–control associations in MACS, corresponding to Supplementary Table 8, including all covariates.

**18**	**MACS SSRI cov**	Full regression models for medication associations in MACS, corresponding to Supplementary Table 10, including all covariates.
**19**	**MACS onlySSRI cov**	Full regression models for SSRI/SNRI specificity analyses in MACS, corresponding to Supplementary Table 12, including all covariates.
**20**	**MACS other meds cov**	Full regression models for non-SSRI/SNRI medication associations in MACS, corresponding to Supplementary Table 12, including all covariates.
**21**	**MACS remission cov**	Full regression models for remission status sensitivity analyses in MACS, corresponding to Supplementary Table 14, including all covariates.
**22**	**MACS somatic cov**	Full regression models for somatic comorbidity sensitivity analyses in MACS, corresponding to Supplementary Table 14, including all covariates.
**23**	**MACS appetite cov**	Full regression models for appetite change sensitivity analyses in MACS, corresponding to Supplementary Table 14, including all covariates.
**24**	**MACS soft drink cov**	Full regression models for soft drink consumption sensitivity analyses in MACS, corresponding to Supplementary Table 14, including all covariates.
**25**	**MACS fiber cov**	Full regression models for fiber intake sensitivity analyses in MACS, corresponding to Supplementary Table 14, including all covariates.
**26**	**MACS calories cov**	Full regression models for total caloric intake sensitivity analyses in MACS, corresponding to Supplementary Table 14, including all covariates.
**27**	**MIND-Set SSRI cov**	Full regression models for medication associations in MIND-Set, corresponding to Supplementary Table 10, including all covariates.
**28**	**MINDSet case-control cov**	Full regression models for MDD–control associations in MIND-Set, corresponding to Supplementary Table 9, including all covariates.
**29**	**MINDSet onlySSRI cov**	Full regression models for SSRI/SNRI specificity analyses in MIND-Set, corresponding to Supplementary Table 12, including all covariates.
**30**	**MIND-Set other meds cov**	Full regression models for non-SSRI/SNRI medication associations in MIND-Set, corresponding to Supplementary Table 12, including all covariates.
**31**	**MINDSet remission cov**	Full regression models for remission status sensitivity analyses in MIND-Set, corresponding to Supplementary Table 14, including all covariates.
