# Supplementary material for: The effect of SSRI/SNRI antidepressant treatment on the gut microbiota of patients with major depressive disorder
Source: Commun Med (Lond). 2026 Jul 16;6:399. doi: 10.1038/s43856-026-01782-5 (PMC13376374; doi:10.1038/s43856-026-01782-5)
Supplement: Supplementary file 1 — Transparent Peer Review file [file 43856_2026_1782_MOESM1_ESM.pdf]

nature portfolio

Peer Review File

The effect of SSRI/SNRI antidepressant treatment on the gut microbiota of patients with major depressive disorder

Corresponding Author: Dr Mirjam Bloemendaal

This file contains all reviewer reports in order by version, followed by all author rebuttals in order by version.

Version 0:

Reviewer comments:

Reviewer #1

(Remarks to the Author)
This study investigates the impact of antidepressant treatment (SSRI/SNRI) on the gut microbiota across multiple cohorts with a relatively large sample size. The finding that the reduction of Clostridium sensu stricto 1 can be explained by SSRI/SNRI treatment status is potentially novel and of interest. However, several aspects of the manuscript require improvement:

1.Baseline characteristics of analytical variables
All variables included in the analyses should have their baseline information presented in Table 1 or supplementary tables. These include, but are not limited to, appetite changes, sampling location, sequencing batch, and sequencing depth.

2.Species-level analysis within Clostridium sensu stricto 1
Can the authors further dissect the composition of species within the Clostridium sensu stricto 1 genus? A species-level differential analysis, accompanied by functional prediction, would provide more mechanistic insight.

3.Drug subclass stratification
Do the available data support additional analyses examining the effects of specific antidepressant subclasses (e.g., individual SSRIs vs. SNRIs) on microbial composition?

4.Subgroup analyses of covariates
The manuscript identifies key taxa after adjusting for covariates. However, further subgroup analyses could clarify whether these covariates independently influence the key taxa and, if so, in what direction and magnitude.

5.Integrated interpretation of key taxa
The discussion should integrate: differences in key microbial abundance between MDD and healthy controls, differences across treatment groups, and functional prediction results alongside relevant literature. Such an integrative analysis would better elucidate the potential role of the identified taxa in the pathophysiology of depression and in the response to antidepressant treatment.

Reviewer #2

(Remarks to the Author)
I co-reviewed this manuscript with one of the reviewers who provided the listed reports. This is part of the Communications Medicine initiative to facilitate training in peer review and to provide appropriate recognition for Early Career Researchers who co-review manuscripts.

Reviewer #3

(Remarks to the Author)
This is an important study that seeks to understand the role of medication use as a mediator of microbial changes in the gut brain access. It puts forward some interesting conclusions. A strength of the paper is the large sample size, which is important. This limits some of the types of biologic analysis. It is important to clarify the limited conclusions to be made using

16s.
The identification of one particular bacteria in the abstract for example feels less important. And its omission does not take away from the paper.
It is also important to discuss challenges with inability to limit by medication burden,, or the contribution of dietary data.

This paper was also reviewed by an early career researcher whom will add updates.

Support publication with minor revisions.

Version 1:

Reviewer comments:

Reviewer #1

(Remarks to the Author)
The authors have attempted the additional analyses suggested by the reviewers. Although the main conclusions of these analyses do not markedly increase the information provided by the manuscript, they have completed the effort based on the available data. The manuscript has fully mined the data and now qualifies for publication. It is hoped that in the future, the authors will further analyze the specific roles of key bacteria in the pharmacotherapy of depression using new data.

Reviewer #2

(Remarks to the Author)
I co-reviewed this manuscript with one of the reviewers who provided the listed reports. This is part of the Communications Medicine initiative to facilitate training in peer review and to provide appropriate recognition for Early Career Researchers who co-review manuscripts.

Reviewer #3

(Remarks to the Author)
No concerns. Publish.

Open Access This Peer Review File is licensed under a Creative Commons Attribution 4.0 International License, which permits use, sharing, adaptation, distribution and reproduction in any medium or format, as long as you give appropriate credit to the original author(s) and the source, provide a link to the Creative Commons license, and indicate if changes were made.
In cases where reviewers are anonymous, credit should be given to 'Anonymous Referee' and the source.
The images or other third party material in this Peer Review File are included in the article’s Creative Commons license, unless indicated otherwise in a credit line to the material. If material is not included in the article’s Creative Commons license and your intended use is not permitted by statutory regulation or exceeds the permitted use, you will need to obtain permission directly from the copyright holder.

To view a copy of this license, visit https://creativecommons.org/licenses/by/4.0/

We appreciate the time and effort that the reviewers have invested in reviewing our manuscript, *'The effect of SSRI/SNRI antidepressant treatment on the gut microbiota of patients with major depressive disorder'.* We have carefully considered and incorporated your comments into the manuscript. Below we elaborate on the reviewers comments point-by-point. All changes in the manuscript are highlighted in green in the revised version of the manuscript, and copied here for convenience. We hope the revisions meet your expectations.

Reviewer #1:

1.Baseline characteristics of analytical variables.
All variables included in the analyses should have their baseline information presented in Table 1 or supplementary tables. These include, but are not limited to, appetite changes, sampling location, sequencing batch, and sequencing depth.
We have added the requested information to **eTable 1**. This table now reports the descriptive statistics for each covariate per MDD and medication group. Furthermore, we have provided more information about sequencing batches, including the number of samples, the sampling location and sequencing depth. To clarify the reviewers comment with regards to "baseline information": MINDSet is a cross-sectional study (only baseline measurements), and MACS is an observational study for which we almost exclusively included information from the baseline measurements. For some individuals in this cohort fecal samples were only available for the follow-up measurement. For these cases, we used the follow-up samples and corresponding clinical information. Hence, all information in the tables refers to the participants information at the time their sample was taken.

Changes in the manuscript (**lines 228-229**):
The full regression models, including covariate effects, are presented in **Supplementary Tables 2**.

2.Species-level analysis within Clostridium sensu stricto 1
Can the authors further dissect the composition of species within the Clostridium sensu stricto 1 genus? A species-level differential analysis, accompanied by functional prediction, would provide more mechanistic insight.

We agree with the reviewer that adding species-level/functional prediction analyses could provide valuable mechanistic insight. In this manuscript we used two datasets for which the V4 region of the 16S rRNA gene was sequenced. Unfortunately this region does not provide sufficient genetic variability to reliably classify up to the species-level[1]. With regards to functional prediction, we are aware of tools such as PICRUSt2, which attempt to perform functional prediction based on 16S data. However, since the latest benchmarks show that 16S-based functional prediction tools generally do not have sufficient sensitivity to identify health-related functional changes in the microbiome, we prefer not to use these[2].

Instead, we attempted to link the 16S rRNA data with already available shotgun metagenomic data for 233 individuals from the MIND-Set cohort (not available for the MACS cohort), derived from the same fecal samples used for 16S rRNA sequencing. Note that for the primary analyses in this manuscript we chose to use the 16S rRNA data, as this was the only sequencing modality available across both cohorts, resulting in a larger combined sample size. The shotgun metagenomic data provided a suitable opportunity to look into the reviewers suggestion of further dissect the composition of species within the *Clostridium sensu stricto 1* genus to gain mechanistic insight.

Shotgun metagenomic sequencing was performed using 2×150 bp paired-end sequencing on the Illumina NovaSeq 6000 platform, with an average sequencing depth of 35.3 million reads

(range: 22.5M–59.4M). Raw sequences were processed into taxonomic profiles using MetaPhlAn4.

MetaPhlAn4 classified a total of 39 species under the *Clostridium* genus (**Rebuttal Table 1**). As a first check, we assessed whether the 16S-derived *Clostridium sensu stricto 1* abundances were associated with the metagenomics-derived *Clostridium* abundances (agglomerated to the genus level). The Pearson correlation was −0.18 (**Rebuttal Figure 1B**), showing a mismatch between what is measured through the 16S' *Clostridium sensu stricto 1* and through MetaPhlAn4's *Clostridium.* We think this is likely explained by the following:

The *Clostridium* genus is known to be highly heterogeneous[3]. Over time, many species have been grouped under *Clostridium* based on morphological similarities rather than genetic relatedness, resulting in a broader, polyphyletic grouping rather than a coherent phylogenetic clade. In recent years, genome-wide comparisons and phylogenetic analyses have led to the reclassification of many of these species into other genera[3], though these updates are not always reflected in reference databases. MetaPhlAn4, despite having its own curated reference database which is based on genetic clustering, still defaults to describing its taxonomy using the traditional clustering as reported in the NBCI taxonomy database[4]. In contrast, the SILVA database adopts genetic similarity-based taxonomy[5], resulting in the smaller *Clostridium sensu stricto 1* genus, which should only contain genetically related species.

As a result, there is an expected mismatch between what MetaPhlan4 considers *Clostridium* (the 39 identified species) and what SILVA considers *Clostridium* (*Clostridium sensu stricto 1*). In an attempt to resolve this, we mapped MetaPhlAn4 species to GTDB taxonomic lineages using the mapping file provided by MetaPhlAn4 (https://zenodo.org/records/15594111). Here we chose GTDB as the intermediary because it clusters based on genetic similarity, and because SILVA itself draws on GTDB as one of its curation resources[5], making GTDB a reasonable common reference point between the two data types. A MetaPhlAn4 species was considered *Clostridium sensu stricto* if it was assigned to both the family Clostridiaceae and the genus *Clostridium* within the GTDB lineage. We acknowledge the limitations of this approach, as it is not a formally validated method for combining 16S rRNA and metagenomic taxonomy, and differences between GTDB and SILVA classifications remain. Nevertheless, in the absence of a standardized framework for integrating these two data types, this mapping represents a pragmatic attempt to place 16S and metagenomic data in the same taxonomic space.

Using this approach, only 10 of the 39 species were retained as (more likely) *Clostridium sensu stricto* 1. The remaining 29 had been reassigned to different genera, including *Enterocloster, Butyribacter*, *Ventrimonas*, *Acetatifactor*, *Copromonas*, *Clostridium_Q* (family Lachnospiraceae), *Clostridium*_AQ (family Erysipelotrichaceae), *Muricomes*, *CHH4-2*, *CAG-127* (family Lachnospiraceae), *Eubacterium*_F, *Lachnoclostridium_A*, *Mediterraneibacter*, *Intestinibacter, and Pseudoruminococcus_A*. **Rebuttal Table 1** shows this MetaPhlAn4-to-GTDB species mapping.

The correlation between 16S-derived *Clostridium sensu stricto 1* and this phylogenetically refined subset of metagenomic species improved to *r*=0.23, but unfortunately remains low (**Rebuttal Figure 1A**). We therefore cannot conclude with certainty that the 16S-derived and metagenomics-derived *Clostridium* are measuring the same organisms.

This discordance appears to be especially pronounced for taxonomically heterogeneous genera such as *Clostridium*. To illustrate this, we assessed the 16S-metagenomics correlation for *Eggerthella*, a genus that has undergone far fewer reclassifications over time. This genus

showed high consistency between the two platforms (r=.873, **Rebuttal Figure 1C**). This suggests that the link between 16S and metagenomic data is present, but challenging to resolve for genera with complex and variable taxonomy like *Clostridium*. This pattern further demonstrates that the mismatch reflects taxonomic nomenclature differences rather than a limitation of the 16S data. Hence, our confidence in the results reported in the manuscript is unaffected.

Taking everything together, given the observed discordance between 16S and metagenomic data for the *Clostridium* genus, we believe the metagenomic data will not provide us with additional mechanistic insight into our 16S-derived findings. For this reason, we are hesitant to carry the metagenomic data forward to differential abundance testing and functional prediction analysis. We share the exploratory work performed on this dataset as part of this rebuttal, but propose to omit it from the manuscript itself. In the discussion we furthermore acknowledge this limitation of drawing mechanistic conclusions based on 16s data alone.

Changes in the manuscript (**lines 333-335**):
The findings from this study depend on 16S-derived taxonomic profiles, limiting the ability to draw conclusions about functional changes. Establishing the functional relevance of these findings will require complementary approaches, including experimental validation and/or metagenomic, metatranscriptomic, or proteomic analyses.

Rebuttal Figure 1. *Scatter plots showing the associations between 16S and metagenomic-derived abundances*

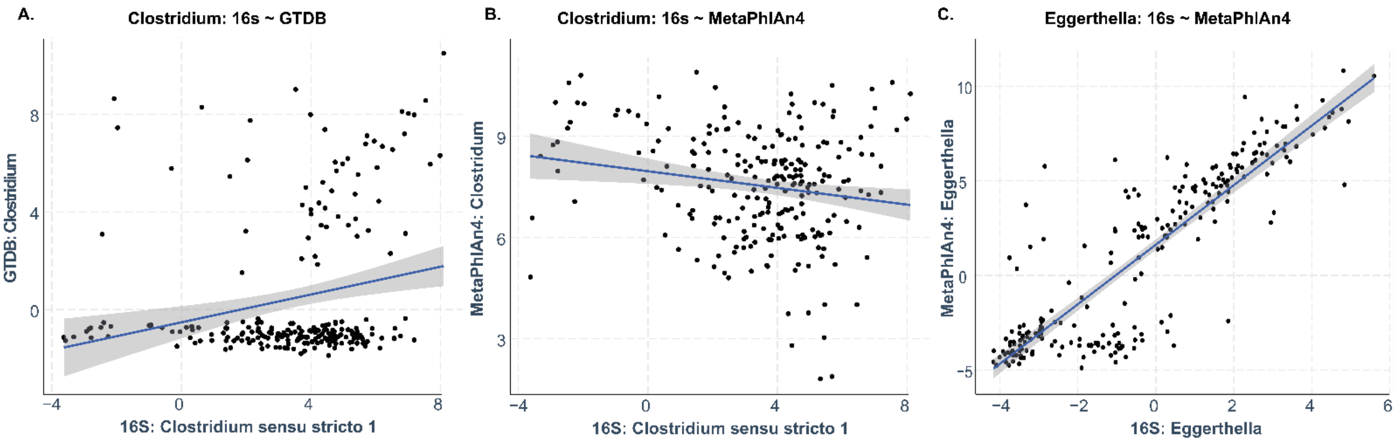

Rebuttal Table 1. *Mapping of MetaPhlAn4 to GTDB*
Species in blue were considered as “sensu stricto” for the phylogenetically refined *Clostridium* subset

MetaPhlAn4 species name	GTDB genus
Clostridium_butyricum	g__Clostridium_AQ|s__Clostridium_AQ innocuum
Clostridium_celatum	g__Clostridium|s__Clostridium celatum
Clostridium_disporicum	g__Clostridium|s__Clostridium disporicum
Clostridium_perfringens	g__Clostridium_P|s__Clostridium_P perfringens
Clostridium_saudiense	g__Clostridium|s__Clostridium saudiense
Clostridium_SGB6173	g__Clostridium|s__Clostridium sp000435835
Clostridium_SGB6177	g__Clostridium|s__Clostridium sp900766315
Clostridium_SGB6179	g__Clostridium|s__Clostridium sp900540255
Clostridium_sp_NSJ_42	g__Clostridium|s__Clostridium sp001916075

Clostridium_thermobutyricum	g__Clostridium|s__Clostridium thermobutyricum
Clostridium_fessum	g__Clostridium_Q|s__Clostridium_Q fessum
Clostridium_SGB26969	g__Acetatifactor|s__
Clostridium_SGB4281	g__Pseudoruminococcus_A|s__Pseudoruminococcus_A sp902765855
Clostridium_SGB4657	g__Butyribacter|s__Butyribacter sp900759665
Clostridium_SGB4750	g__Enterocloster|s__Enterocloster sp900541315
Clostridium_SGB4751	g__Enterocloster|s__Enterocloster sp900543885
Clostridium_SGB48024	g__Ventrimonas|s__Ventrimonas sp900770535
Clostridium_SGB4909	g__Acetatifactor|s__Acetatifactor sp003447295
Clostridium_sp_1001270H_150608_G6	g__CAG-127|s__CAG-127 sp900319515
Clostridium_sp_1001270J_160509_D11	g__Intestinibacter|s__Intestinibacter sp900540355
Clostridium_sp_1001271st1_H5	g__Enterocloster|s__Enterocloster sp005845215
Clostridium_sp_AF12_28	g__Copromonas|s__Copromonas sp900066055
Clostridium_sp_AF20_17LB	g__Copromonas|s__Copromonas sp900066785
Clostridium_sp_AF27_2AA	g__Ventrimonas|s__Ventrimonas sp003478505
Clostridium_sp_AF32_12BH	g__Ventrimonas|s__Ventrimonas sp003481825
Clostridium_sp_AF34_10BH	g__Acetatifactor|s__Acetatifactor sp900066565
Clostridium_sp_AF34_13	g__Butyribacter|s__Butyribacter intestini
Clostridium_sp_AF36_4	g__Eubacterium_F|s__Eubacterium_F sp003491505
Clostridium_sp_AM22_11AC	g__Enterocloster|s__Enterocloster sp000431375
Clostridium_sp_AM33_3	g__Ventrimonas|s__Ventrimonas sp003480315
Clostridium_sp_AM42_4	g__Ventrimonas|s__Ventrimonas sp003506385
Clostridium_sp_AM49_4BH	g__Butyribacter|s__Butyribacter sp003529475
Clostridium_sp_AM58_1XD	g__Lachnoclostridium_A|s__Lachnoclostridium_A sp003464085
Clostridium_sp_AT4	g__Enterocloster|s__Enterocloster sp001517625
Clostridium_sp_C5_48	g__Enterocloster|s__Enterocloster sp900538485
Clostridium_sp_chh4_2	g__CHH4-2|s__CHH4-2 sp002899675
Clostridium_sp_D5	g__Muricomes|s__Muricomes fissicatena_A
Clostridium_sp_Marseille_P3244	g__Mediterraneibacter|s__Mediterraneibacter sp900120155
Clostridium_sp_OM07_10AC	g__Butyribacter|s__Butyribacter sp001916135

3.Drug subclass stratification

Do the available data support additional analyses examining the effects of specific antidepressant subclasses (e.g., individual SSRIs vs. SNRIs) on microbial composition?
Evidence from in vitro screening indeed indicates that microbial susceptibility can differ between individual compounds[6–8], and we agree that subclass analyses would be a very interesting addition to the paper. Unfortunately, the available data does not support additional subgroup analyses, as the numbers per specific SSRI/SNRI are small and highly unbalanced (see **Figure 2B,D** for sample sizes per drug type). For example, Venlafaxin (the most commonly used SSRI/SNRI) is used by 93 individuals in the MACS cohort, Citalopram, the second most common, by 66, and Escitalopram, the third most common by 53. All other medications, are used by fewer than 50 individuals (range: 4-44). In the MIND-Set cohort Citalopram was the most commonly used SSRI/SNRI, used by 17 participants. As already noted in the Discussion (**lines 356-361**), further stratification by compound would therefore result in underpowered comparisons and a high risk of spurious findings.

To confirm this, we conducted a power analysis based on the observed log OR (−0.308) for the association between *Clostridium sensu stricto 1* abundance and SSRI/SNRI treatment. We used the *powerLogisticCon* function (powerMediation package in R), which calculates power for logistic regression with a continuous predictor. The harmonic mean of the two group sizes

(unmedicated vs. medication subgroup) was used as the effective n per group, to handle unequal group sizes[9]. For the medication subgroup analyses, the power would range from 0.089 (Milnacipran, n = 4) to 0.739 (Venlafaxin, n = 93), which is below the conventional 80% threshold. These estimates are likely optimistic, as they do not account for covariate adjustment or the stricter significance threshold required for multiple testing correction, both of which would reduce power further.

For this reason we chose to focus on class-level SSRI/SNRI effects in the current manuscript. The sensitivity analyses presented demonstrated that the identified associations were specific to SSRI/SNRIs and not generalizable to other classes of psychotropic medications (section ***SSRI/SNRI specificity***, **lines 261-273** and **eTable 10**). We agree that future research, with sufficient power, should perform drug-type specific analyses to further disentangle the effects.

4.Subgroup analyses of covariates
The manuscript identifies key taxa after adjusting for covariates. However, further subgroup analyses could clarify whether these covariates independently influence the key taxa and, if so, in what direction and magnitude.
We agree that covariate-specific subgroup analyses could be of interest. Unfortunately, the cohorts examined in this work do not provide sufficient statistical power to address these questions reliably (see power calculations described under point 3). That said, to provide more transparency regarding covariate effects in our models, we added the complete summary statistics, including all covariates, for every regression model reported in this work (see **Supplementary Tables 2**). We also added a elaboration on the covariate effects for the association between *Clostridium sensu stricto 1* and SSRI/SNRI treatment

Changes in manuscript: (**lines 243-246**):
None of the other covariates (age, sex, BMI, anxiety symptoms, depression symptoms, collection site and sequencing depth,) were significantly associated with *Clostridium sensu stricto 1* abundance in the quantile regression model (**Supplementary Tables 2)**.

5.Integrated interpretation of key taxa
The discussion should integrate: differences in key microbial abundance between MDD and healthy controls, differences across treatment groups, and functional prediction results alongside relevant literature. Such an integrative analysis would better elucidate the potential role of the identified taxa in the pathophysiology of depression and in the response to antidepressant treatment.

In response to the reviewer's comment, we have revised the first paragraph of the Discussion to provide a more integrated interpretation of the associated genera. At the same time, the primary aim of this study is to investigate the impact of SSRI/SNRI treatment on the gut microbiome rather than to characterize microbial contributions to MDD pathophysiology. We therefore decided to maintain a treatment-focused interpretation on the findings throughout the Discussion. This includes functional context directly related to the medication-associated genus *Clostridium sensu stricto 1*, while avoiding broader mechanistic inferences about the pathophysiology of depression

Changes in manuscript (**lines 286-298**):
In this study, we aimed to identify the extent to which SSRIs and SNRIs modify gut microbial differences associated with MDD, thereby distinguishing disorder-related signals from those more likely shaped by pharmacological treatment. We identified nine genera with differential abundance between MDD patients and controls, including elevated *Eggerthella* and *Hungatella*. This replicates findings in a largely overlapping sub-sample of the MACS cohort

and from independent studies, especially the latter underscoring their consistency as markers of the disorder. Among these taxa, *Clostridium sensu stricto 1* showed a distinct pattern in relation to medication: its reduced abundance in MDD patients appeared to be driven by SSRI/SNRI treatment, with lower levels observed in treated compared to unmedicated patients, and its association with MDD diagnosis only evident in the treated group. This identifies *Clostridium sensu stricto 1* as a potentially medication-sensitive genus, while the other MDD associated genera, which were not affected by medication status, may reflect other disorder-related factors instead, such as host genetics of lifestyle.

Reviewer #2-3:

It is important to clarify the limited conclusions to be made using 16s.
We have added a disclaimer about speculation about function based on 16S data.

Changes in the manuscript (**lines 333-335**):
The findings from this study depend on 16S-derived taxonomic profiles, limiting the ability to draw conclusions about functional changes. Establishing the functional relevance of these findings will require complementary approaches, including experimental validation and/or metagenomic, metatranscriptomic, or proteomic analyses.

The identification of one particular bacteria in the abstract for example feels less important. And its omission does not take away from the paper.
We thank the reviewer for this comment. While we acknowledge that medication likely exerts a broad, modest effect across a range of gut bacteria, we believe that the replicated association of *Clostridium sensu stricto 1* serves as a statistically robust example of a medication-driven signal in our data, directly illustrating how treatment confounds the MDD association. As such, this finding is central to the manuscript's message, and we have decided to retain it in the abstract.

It is also important to discuss challenges with inability to limit by medication burden
We think the reviewer means that medication burden (i.e., side effects, number of medications, dose and/or duration of use) can be a modifier of the effect we observe and we agree that medication burden may contribute to the observed microbial differences. Unfortunately, information on medication dose, treatment duration, and other medication-related factors was not collected in a systematic way, restricting our ability to perform more extensive analyses on this. That said, the observed reduction in *Clostridium sensu stricto 1* was specific to SSRI/SNRI users and was not replicated in users of other psychotropic medications, suggesting the association is unlikely to be fully explained by general medication burden alone. We have added this point as a limitation in the Discussion.

Changes in the manuscript (**lines 352-356**):
Second, limited information on side effects, medication dosage, comedication and treatment duration, restricted our ability to assess the effect of medication burden. However, given that observed associations were not replicated in patients treated with non-SSRI/SNRI psychotropics, it seems unlikely that the signal is explained by general medication burden alone.

It is also important to discuss or the contribution of dietary data.
We realize that the description of the dietary results in the main text was not comprehensive enough to follow, and we thank the reviewer for flagging this. We have elaborated on this in the main text of the updated manuscript, adding details about the size of the tested subset, which dietary factors were corrected for, and the effect sizes with significance.

Updated paragraph in the manuscript (**lines 277-283**):
Dietary adjustment (fiber intake, caloric intake, and soft drink consumption) was possible in a subset of participants from the MACS cohort where these data were available (N=222). Effect sizes remained comparable to the unadjusted models (**Supplementary Results; eTable 12**), suggesting that dietary differences between treatment groups do not explain the observed reduction in *Clostridium sensu stricto 1*. Associations were, however, no longer statistically significant, which is likely attributable to the reduced sample size than confounding by diet.

References

1. Johnson, J. S. *et al.* Evaluation of 16S rRNA gene sequencing for species and strain-level microbiome analysis. *Nat Commun* **10**, 5029 (2019).
2. Matchado, M. S. *et al.* On the limits of 16S rRNA gene-based metagenome prediction and functional profiling. *Microbial Genomics* **10**, 001203 (2024).
3. Cruz-Morales, P. *et al.* Revisiting the Evolution and Taxonomy of Clostridia, a Phylogenomic Update. *Genome Biol Evol* **11**, 2035–2044 (2019).
4. Blanco-Míguez, A. *et al.* Extending and improving metagenomic taxonomic profiling with uncharacterized species using MetaPhlAn 4. *Nat Biotechnol* **41**, 1633–1644 (2023).
5. Chuvochina, M. *et al.* SILVA in 2026: a global core biodata resource for rRNA within the DSMZ digital diversity. *Nucleic Acids Res* **54**, D334–D341 (2025).
6. Kurilshikov, A. *et al.* Large-scale association analyses identify host factors influencing human gut microbiome composition. *Nat Genet* **53**, 156–165 (2021).
7. Cussotto, S., Clarke, G., Dinan, T. G. & Cryan, J. F. Psychotropics and the Microbiome: a Chamber of Secrets…. *Psychopharmacology* **236**, 1411–1432 (2019).
8. Maier, L. *et al.* Extensive impact of non-antibiotic drugs on human gut bacteria. *Nature* **555**, 623–628 (2018).
9. Konstantopoulos, S. Power Analysis in Two-Level Unbalanced Designs. *The Journal of Experimental Education* **78**, 291–317 (2010).
